# A UV-LED module that is highly effective at inactivating human coronaviruses and HIV-1

**DOI:** 10.1186/s12985-022-01754-w

**Published:** 2022-02-10

**Authors:** Arvin T. Persaud, Jonathan Burnie, Laxshaginee Thaya, Liann DSouza, Steven Martin, Christina Guzzo

**Affiliations:** 1 Department of Biological Sciences, University of Toronto Scarborough, 1265 Military Trail, Room SW560, Toronto, ON M1C 1A4 Canada; 2 Department of Cell and Systems Biology, University of Toronto, 25 Harbord Street, Toronto, ON M5S 3G5 Canada; 3 Safe Antiviral Technologies Inc, 822 Manning Ave, Toronto, ON M6G 2W8 Canada

**Keywords:** Ultraviolet light (UV), Light emitting diode (LED), Virus inactivation, UV sanitization, UV disinfection, *Bacillus pumilus*, Human coronavirus (hCoV), Human immunodeficiency virus (HIV)

## Abstract

Ultraviolet (UV) light has previously been established as useful method of disinfection, with demonstrated efficacy to inactivate a broad range of microorganisms. The advent of ultraviolet light-emitting diodes provides advantages in ease of disinfection, in that there can be delivery of germicidal UV with the same light unit that delivers standard white light to illuminate a room. Herein we demonstrate the efficacy and feasibility of ultraviolet light-emitting diodes as a means of decontamination by inactivating two distinct virus models, human coronavirus 229E and human immunodeficiency virus. Importantly, the same dose of ultraviolet light that inactivated human viruses also elicited complete inactivation of ultraviolet-resistant bacterial spores (*Bacillus pumilus*), a gold standard for demonstrating ultraviolet-mediated disinfection. This work demonstrates that seconds of ultraviolet light-emitting diodes (UV-LED) exposure can inactivate viruses and bacteria, highlighting that UV-LED could be a useful and practical tool for broad sanitization of public spaces.

## Introduction

Disinfection of fomites remains an important public health measure in reducing the spread of a variety of communicable diseases, especially during an ongoing pandemic. With recent recognition of the roles close contact and indoor crowding have in virus transmission [1, 2], there is certainly an interest in developing technologies that would provide for frequent, high-throughput disinfection, especially of highly trafficked public spaces.

Conventional chemical disinfectants used in clinical and laboratory spaces, while effective, are an impractical option to deploy on a large scale due to the environmental, public health, and infrastructural hazards associated with the active ingredients. Additionally, the use of chemical disinfectants includes variability in efficacy contributed by the user and their attention to reproducible and prudent cleaning protocols. As an alternative, ultraviolet (UV) radiation can be automated to deliver a reproducible germicidal dose, and has been extensively used for inactivating various microbes, including several types of viruses [3–10]. The development of UV light emitting diodes (LEDs) offers the same level of decontamination as with the conventional mercury lamps, but with several advantages [11, 12], including ease of retrofit in a range of typical over-head light sources, with added disinfection capabilities. The usefulness of UV for disinfection is underscored by its simple mechanism of action. The nucleotide bases of DNA and RNA absorb UV photons but uniquely, adjacent thymine bases (or uracil, in the case of RNA) undergo dimerization, disrupting the structure of nucleotide sequences and introducing ‘roadblocks’ in genome replication [13].

Here we demonstrate the antiviral efficacy of a UV-LED module by inactivating two distinct viruses, the seasonal human coronavirus 229E (hCoV-229E) and human immunodeficiency virus type 1 (HIV-1). Using a droplet dispersal method to mimic typical environmental occurrences of virus shedding (e.g., sneeze, cough, blood droplets), we show significant reductions in viral replication within seconds of UV-LED exposure. Our work adds to the growing literature on the application of UV-LEDs for the disinfection of high-contact public spaces. Given that UV-LEDs are low cost and easily installed into a variety of existing light fixtures, they represent an additional, highly efficacious layer of protection against pathogen spread, particularly in times of an ongoing respiratory virus pandemic.

## Materials and methods

### Cell culture and viruses

The TZM-bl cell line was obtained from the NIH AIDS Reagent Program (cat# ARP-8129) and maintained in high-glucose (4.5 g L^−1^) DMEM (Wisent cat# 319005-CL). MRC-5 was maintained in EMEM (ATCC cat# 30-2003) and Huh7 in low-glucose (1 g L^−1^) DMEM (Gibco cat# 11885-084). All media were supplemented with 10% (v/v) heat-inactivated fetal bovine serum (FBS; Wisent cat# 098150), 100 U mL^−1^ penicillin and 100 µg mL^−1^ streptomycin (Fisher Scientific cat# 15140122), and all cell lines were kept at 37 °C in a humidified, 5% CO_2_ incubator.

HIV-1 IIIB stocks were generated through infection of the A3R5.7 T cell line (NIH ARP cat# 12386). In brief, cells were pelleted and resuspended in 1 mL of HIV-1 IIIB isolates for 4 h before fresh media was added. Virus-containing supernatants were harvested 10–12 days later, aliquoted, and frozen at − 80 °C. To quantify virus concentration and standardize input for infection assays, HIV-1 p24 capsid protein levels were measured using the AlphaLISA p24 detection kit following the manufacturer’s (PerkinElmer) instructions. Absorbance readings were performed on a Synergy NEO 2 multimode plate reader (BioTek) equipped with Gen 5 software (v. 3.08).

Wild-type (hCoV-229E) and EGFP-expressing (229E-EGFP) human coronavirus 229E were produced by infecting MRC-5 cells for 48 h (hCoV-229E) or Huh7 cells for 72 h (229E-EGFP) at 34 °C after which cell-free supernatants were aliquoted and stored at − 80 °C. Both virus stocks were titrated on Huh7 cells (2 × 10^4^ cells/well; 96-well plate) by overlaying 50 µL of 1:10 serial dilutions of virus-containing supernatants in serum-free DMEM for a 1 h adsorption at 34 °C, as similarly described [14]. Following, inoculum was removed and replaced with 200 µL DMEM + 2% (v/v) FBS and infection was monitored for 5 days at 34 °C. TCID_50_ calculations based on observable cytopathic effect were done as previously described [15].

### UV-LED specifications

The UV-LEDs were supplied as two sets, nine 275 nm LEDs in a 3 × 3 array, and twenty 380 nm LEDs in a 4 × 5 array. The LEDs were approximately 5 cm from the irradiated sample, with each array delivering between 0.4 and 0.6 mW/cm^2^ of UV light. The maximum irradiation time was 30 s, resulting in a total delivered dose for the combined arrays of 8 mJ/cm^2^ to 20 mJ/cm^2^ to the irradiated samples. The area illuminated was substantially larger than the irradiated sample, with the total lit area of the device approximately 10 cm by 20 cm, or a total of 200 cm^2^, resulting in a total areal dose of 1.6 J to 4 J.

### *Bacillus pumilus* inactivation assay

Stainless steel discs inoculated with *Bacillus pumilus* spores were obtained through Mesa Labs (cat# DPSSC/3). Discs were exposed on both sides to the UV light for the specified times and cultured in tryptic soy both (Fisher Scientific cat# DF0370-17-3). Cultures were incubated for seven days at 33 °C under aerobic conditions. For turbidity measurements, samples were transferred into 96-well plates (FroggaBio cat# 92096) in duplicate and optical density (OD, 600 nm) measurements were determined using the Synergy Neo2 multimode plate reader. Final data presentation was done on Prism (GraphPad, v.9.1.2) for all experiments.

### HIV-1 inactivation assay

For UV exposure, stock virus (82 ng mL^−1^ p24) was diluted and dispersed into 7 µL droplets, exposed to UV for 30 s, and then re-pooled for assaying by TZM-bl titration. TZM-bl cells were overlaid at 1.5 × 10^4^ cells/well over the virus dilutions in 96-well plates. After 4 days of culture, cells were lysed and incubated in BriteLite Plus (Perkin Elmer cat# 6066766) for 10 min at room temperature, transferred to opaque white Opti Plates (Perkin Elmer) for luminescence read-out on the Synergy Neo2 multimode plate reader equipped with Gen5 (v.3.08) (BioTek).

### hCoV-229E inactivation assays

For UV exposure of wild-type hCoV-229E, samples were prepared in serum-free DMEM (MOI 0.1, 0.01 and 0.0001), distributed in 7 µL droplets and exposed to UV for 30 s. Following exposure, virus droplets were picked up and diluted to 700 µL in serum-free DMEM. The entire inoculum was overlaid onto monolayers of 6 × 10^5^ Huh7 cells plated in 6-well plates 24 h prior. Inoculated cells were incubated for 1 h at 34 °C to allow for adsorption before DMEM + 2% (v/v) FBS was added for a final volume of 2 mL/well. After 48 h of infection, monolayers were trypsinized, harvested and total RNA was extracted using the GENEzol TriRNA kit (Geneaid Biotech cat# 12183020). RNA purity and quantitation were done on the BioTek’s Synergy Neo2 multimode plate reader using the Take3 adapter. Real-time PCR was done using the TaqMan Fast Virus 1-Step Master Mix (Thermo Fisher cat# 4444432). Optimized primer–probe TaqMan assays were obtained from Thermo Fisher for the detection of eukaryotic 18S rRNA (cat# 4453320, assay ID: Hs99999901_s1) and hCoV-229E (cat# 4331182, assay ID: Vi06439671_s1). PCR was performed on the Quant Studio 3 and Thermo Fisher’s cloud-based Relative Quantitation module was used for data analysis.

For experiments with EGFP-expressing hCoV-229E, stock virus was diluted 1:30 (akin to MOI 0.01) and irradiated as described above and used to infect monolayers of 2 × 10^4^ Huh7 cells in 96-well plates. Inoculated cells were incubated for 1 h at 34 °C to allow for adsorption before DMEM + 2% (v/v) FBS was added for a final volume of 0.2 mL/well. After 72 h of infection, cells were collected, fixed (2% v/v PFA) and GFP fluorescence was analyzed on the BD LSR Fortessa, with analyses performed in FlowJo (v.10.7.2).

## Results

**Fig. 1:**
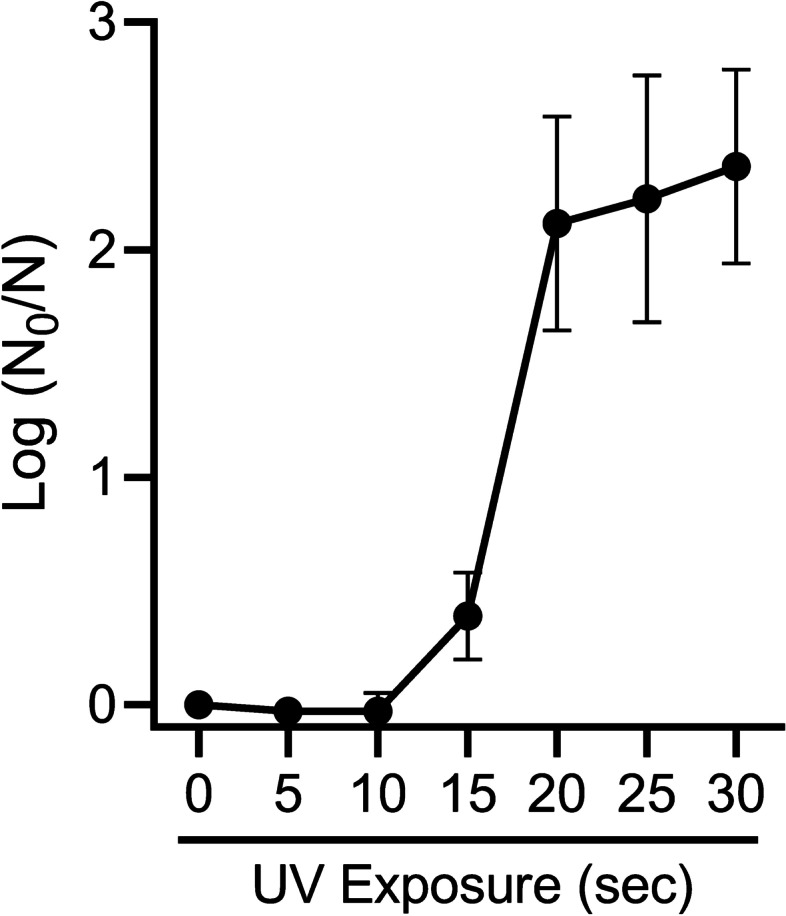
Kinetic analysis of *Bacillus pumilus* growth after UV exposure*.* Stainless steel discs inoculated with *Bacillus pumilus* spores were exposed to UV for the specified times after which the discs were used to inoculate liquid cultures. After seven days in culture, optical density (OD, 600 nm) readings were taken. Log-reduction was calculated by taking the base-10 logarithm of the quotient (N_0_/N), where N_0_ and N represent the untreated and irradiated discs, respectively. Data are mean ± SD of four experimental replicates across two independent experiments. OD measurements were done in triplicates

To first establish the efficacy of the UV-LED, we inactivated *B. pumilus* spores, which are recognized as a standard biological indicator for sterilization by ionizing radiation. Due to the well characterized resistance to UV radiation [16–18], *B. pumilus* can be used in UV disinfection studies as a surrogate organism for UV-resistant pathogens [19]. A kinetic analysis evaluating different UV-LED exposure times revealed that bacterial growth was reduced with as little as 15 s of exposure, but a > 2-Log (99%) reduction required at least 20 s of UV exposure (Fig. 1). For reference, the level of turbidity seen for discs irradiated > 20 s was comparable to the turbidity of negative controls (medium only) and discs that were autoclaved (data not shown).

**Fig. 2:**
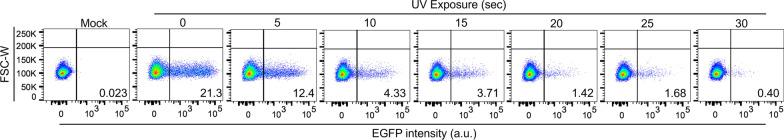
Kinetic analysis of hCoV-229E infection after UV exposure*.* hCoV-229E-EGFP was exposed to UV for varying times and susceptible cells were infected for 3 days after which they were collected and analyzed by flow cytometry for EGFP fluorescence (FITC filter). FSC-W vs EGFP plots show the EGFP + cells in lower right quadrant, with the percent positive cells annotated. Data shown are representative of three experimental replicates with similar results

We anticipated that the kinetics of UV-mediated inactivation observed on UV-resistant bacterial spores may be different on replicating viruses, therefore we evaluated the kinetic inactivation of the seasonal human coronavirus 229E (hCoV-229E). We utilized a recombinant hCoV-229E in which ORF4 was replaced with GP-EGFP [20, 21] (229E-EGFP, henceforth), permitting EGFP fluorescence as a readout for infection. Flow cytometric analysis of Huh7 cells evaluated three days post-infection revealed a reproducible fraction of infected cells in the absence of UV-LED exposure (Fig. 2, 21.3% EGFP + events at 0 s). With increasing exposure to the UV-LED, the proportion of infected cells decreased substantially, to just 0.4% of cells at 30 s of exposure, similar to the proportion background GFP-positive cells observed in the Mock infected sample (0.023%).

**Fig. 3:**
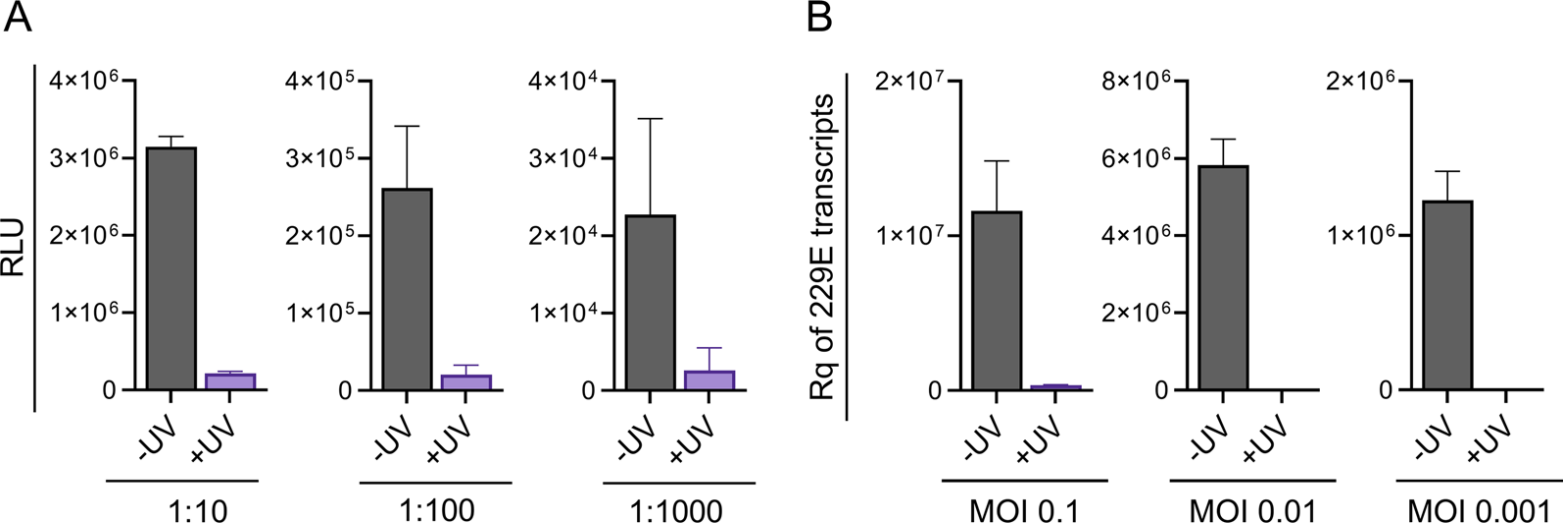
UV effectively inactivates viruses of varying titres*.* **A** Dilutions of HIV-1 IIIB was left untreated (UV-, grey bars), irradiated with UV (UV+, purple bars) for 30 s. Virus replication post-treatment was determined by measuring luminescence (RLU, relative light units) of a luciferase-based reporter cell line. Bars are mean ± SD of duplicate luminescence readings and are representative of three independent experiments. **B** Three MOIs of hCoV-229E were left untreated (UV-, grey bars) or irradiated with UV for 30 s (UV+, purple bars). Virus replication was determined by measuring the relative quantity (Rq) of hCoV-229E transcripts by qPCR in total RNA isolated from infected cells. Eukaryotic 18S rRNA was used for the endogenous control and mock-infected cells was used as the reference sample for Rq. Bars are mean ± SD of triplicate qPCR assays and are representative of two independent experiments

Next, we assessed the ability of UV-LED to inactivate a range of virus titres, using human immunodeficiency virus type I (HIV-1) as an additional virus model here. HIV-1 is an enveloped human retrovirus with a genome consisting of two identical strands of RNA enclosed in a nucleocapsid core [22]. We tested three levels of input HIV-1 that were serially diluted tenfold, observed with a luminescence intensity that is roughly tenfold apart in Fig. 3A (UV-negative bars in grey). Contrastingly, with 30 s of UV exposure (UV+), infectivity was markedly reduced (Fig. 3A, purple bars); 93%, 92% and 88% reduction for the 1:10, 1:100 and 1:1000 virus dilutions, respectively. We similarly assessed the inactivation of various titres of wild-type hCoV-229E to determine if the MOI-dependent differences of UV inactivation can be reproduced using an alternative virus model. Following the presence/absence of UV treatment for 30 s, total RNA was isolated from infected cells 48 h post-infection and probed for hCoV-229E transcripts by qPCR. Viral replication was effectively reduced after exposure to UV as shown by the decreased hCoV-229E transcripts (Fig. 3B, grey untreated vs purple UV-treated bars), representing a 1.5-, 5.5- and 5.8-Log reduction for MOIs 0.1, 0.01 and 0.001, respectively. This likely reflects an MOI-dependent susceptibility to UV inactivation, in that high-titre viral samples are more difficult to inactivate than samples with fewer viral particles. These larger differences were not readily apparent with the HIV experiments (Fig. 3A), presumably due to the use of the TZM-bL reporter assay used to evaluate infection, which includes a consistent background luminescence (RLU) from the reporter cells used [23].

## Conclusions

Taken together, this work demonstrates the effectiveness of a UV-LED module as a means of decontamination by inactivating (1) UV-resistant bacterial spores, (2) HIV-1 and (3) the human coronavirus 229E, observing up to a 5.8-Log reduction in viral replication, in the case of the human coronavirus. While we did not directly assess whether this reduction in hCoV-229E replication correlates with a similar reduction in infectivity, we do anticipate a similar reduction in infectivity after exposure to UV, given that the specific mechanism of inactivation by UV irradiation is RNA damage and hCoV-229E is an RNA virus. In this study we acknowledge that our selected virus models were all enveloped viruses, chosen to assess any potential differences in UV susceptibility due to viral genome length [24], and we therefore caution that our results cannot be directly applied to assess efficacy in disinfecting non-enveloped virus, as they are generally more resistant to UV than enveloped viruses. However, we believe that our experiments showing inactivation of the *B. pumilus* spores, known for their high level of UV resistance, can provide a first indication for UV inactivation of non-enveloped viruses. Indeed, spores of *B. pumilus* have been suggested as a surrogate for evaluating the inactivation of the non-enveloped human adenovirus by UV irradiation [19]. Importantly, additional studies using non-enveloped virus models are needed to assess the antiviral activity of UV-LED beyond the enveloped viruses tested in this study.

Contaminated fomites [25] and indoor crowding [1, 2] remain important routes of transmission for a variety of communicable diseases, including SARS-CoV-2 and other respiratory pathogens. Routine disinfection of high-contact, public spaces may mitigate the risks of transmission. UV-LEDs provide the advantage of being flexibly integrated into existing light fixtures, allowing automated sanitization of a variety of different spaces, including offices, malls, gyms, and public transit. Although we only observed a 2–2.5 log-reduction in the bacterial spores, we maintain that this platform is a useful adjunct to existing prevention methods, and one that can easily be implemented at a low cost. While complete sterilization in public spaces may not be an attainable goal, routine disinfection of high traffic spaces with iterative UV exposures throughout the day can mitigate the risks associated with fomite transmission of infectious diseases. Our work adds to the growing literature on the application of UV-LEDs for disinfection and highlights that this cost-effective prevention method may be an important and practical adjunct to existing disinfection strategies to minimize transmission of communicable diseases in public places.

## Data Availability

The datasets used and/or analysed during the current study are available from the corresponding author on reasonable request.

## Abbreviations

HIV: Human immunodeficiency virus
hCoV: Human coronavirus
UV: Ultraviolet
LED: Light-emitting diode
GFP: Green fluorescent protein
